# The “inconvenient truth” about AI in healthcare

**DOI:** 10.1038/s41746-019-0155-4

**Published:** 2019-08-16

**Authors:** Trishan Panch, Heather Mattie, Leo Anthony Celi

**Affiliations:** 1 Division of Health Policy and Management, Harvard T.H. Chan School of Public Health, Boston, MA USA; 2 Wellframe Inc., Boston, MA USA; 3 Department of Biostatistics, Harvard T.H. Chan School of Public Health, Boston, MA USA; 4 Institute for Medical Engineering and Science, Massachusetts Institute of Technology, Cambridge, MA USA; 5 Division of Pulmonary, Critical Care and Sleep Medicine, Beth Israel Deaconess Medical Center, Boston, MA USA

**Keywords:** Health policy, Public health

As the UK sits in painful deadlock over Brexit, it is important to remember that governments are regularly faced with crises, and their responses can create enduring benefit for future generations. Back in 1858, for example, the UK parliament was dealing with another messy crisis: “the great stink.” In a world before sanitation, the river Thames had become an open latrine, and as summer blossomed parliament was engulfed in a pestilential stench. £2.5 million (about £300 million in today’s money) was hastily approved to build a network of sewers throughout the capital.^1^ This particular model of sanitation, developed by Bazalgette, was adopted by other cities around the world and the rest, as they say, is history. It is now unthinkable that a developed nation would not have sanitation infrastructure. However, back in 1858 the debate was whether sanitation infrastructure was worthy of investment and whether it was a public or private good. A similar debate has been simmering for some time regarding health data infrastructure, defined as the hardware and software to securely aggregate, store, process and transmit healthcare data. Is data infrastructure necessary for healthcare organizations and if so, is it the responsibility of individual healthcare organizations, of local health systems, or is it a public good?

In the 21st Century, the age of big data and artificial intelligence (AI), each healthcare organization has built its own data infrastructure to support its own needs, typically involving on-premises computing and storage.^2,3^ Data is balkanized along organizational boundaries, severely constraining the ability to provide services to patients across a care continuum within one organization or across organizations. This situation evolved as individual organizations had to buy and maintain the costly hardware and software required for healthcare, and has been reinforced by vendor lock-in, most notably in electronic medical records (EMRs). With increasing cost pressure and policy imperatives to manage patients across and between care episodes, the need to aggregate data across and between departments within a healthcare organization and across disparate organizations has become apparent not only to realize the promise of AI but also to improve the efficiency of existing data intensive tasks such as any population level segmentation^4^ and patient safety monitoring.^5^

The rapid explosion in AI has introduced the possibility of using aggregated healthcare data to produce powerful models that can automate diagnosis^6^ and also enable an increasingly precision approach to medicine by tailoring treatments and targeting resources with maximum effectiveness in a timely and dynamic manner.^7,8^

However, “the inconvenient truth” is that at present the algorithms that feature prominently in research literature are in fact not, for the most part, executable at the frontlines of clinical practice. This is for two reasons: first, these AI innovations by themselves do not re-engineer the incentives that support existing ways of working.^2^ A complex web of ingrained political and economic factors as well as the proximal influence of medical practice norms and commercial interests determine the way healthcare is delivered. Simply adding AI applications to a fragmented system will not create sustainable change. Second, most healthcare organizations lack the data infrastructure required to collect the data needed to optimally train algorithms to (a) “fit” the local population and/or the local practice patterns, a requirement prior to deployment that is rarely highlighted by current AI publications, and (b) interrogate them for bias to guarantee that the algorithms perform consistently across patient cohorts, especially those who may not have been adequately represented in the training cohort.^9^ For example, an algorithm trained on mostly Caucasian patients is not expected to have the same accuracy when applied to minorities.^10^ In addition, such rigorous evaluation and re-calibration must continue after implementation to track and capture those patient demographics and practice patterns which inevitably change over time.^11^ Some of these issues can be addressed through external validation, the importance of which is not unique to AI, and it is timely that existing standards for prediction model reporting are being updated specifically to incorporate standards applicable to this end.^12^ In the United States, there are islands of aggregated healthcare data in the ICU,^13^ and in the Veterans Administration.^14^ These aggregated data sets have predictably catalyzed an acceleration in AI development; but without broader development of data infrastructure outside these islands it will not be possible to generalize these innovations.

Elsewhere in the economy, the development of cloud computing, secure high-performance general use data infrastructure and services available via the Internet (the “cloud”), has been a significant enabler for large and small technology companies alike, providing significantly lower fixed costs and higher performance as well as supporting the aforementioned opportunities for AI. Healthcare, with its abundance of data, is in theory well-poised to benefit from growth in cloud computing. The largest and arguably most valuable store of data in healthcare rests in EMRs. However, clinician satisfaction with EMRs remains low, resulting in variable completeness and quality of data entry, and interoperability between different providers remains elusive.^11^ The typical lament of a harried clinician is still “why does my EMR still suck and why don’t all these systems just talk to each other?” Policy imperatives have attempted to address these dilemmas, however progress has been minimal. In spite of the widely touted benefits of “data liberation”,^15^ a sufficiently compelling use case has not been presented to overcome the vested interests maintaining the status quo and justify the significant upfront investment necessary to build data infrastructure. Furthermore, it is reasonable to suggest that such high-performance computing work has been and continues to be beyond the core competencies of either healthcare organizations or governments^16^ and as such, policies have been formulated, but rarely, if ever, successfully implemented. It is now time to revisit these policy imperatives in light of the availability of secure, scalable data infrastructure available through cloud computing that makes the vision of interoperability realizable, at least in theory.

To realize this vision and to realize the potential of AI across health systems, more fundamental issues have to be addressed: who owns health data, who is responsible for it, and who can use it? Cloud computing alone will not answer these questions—public discourse and policy intervention will be needed. The specific path forward will depend on the degree of a social compact around healthcare itself as a public good, the tolerance to public private partnership, and crucially, the public’s trust in both governments and the private sector to treat their healthcare data with due care and attention in the face of both commercial and political perverse incentives.

In terms of the private sector these concerns are amplified as cloud computing is provided by a small number of large technology companies who have both significant market power and strong commercial interests outside of healthcare for which healthcare data might potentially be beneficial. Specific contracting instruments are needed to ensure that data sharing involves both necessary protection as well as, where relevant, fair material returns to healthcare organizations and the patients they serve.^17^ In the absence of a general approach to contracting, high profile cases in this area have been corrosive to public trust.^18,19^ Data privacy regulations like the European Union’s General Data Protection Regulation^20^ (GDPR) or California’s Consumer Privacy Act^21^ are necessary and well intentioned, though incur the risk of favoring well-resourced incumbents who are more able to meet the cost of regulatory compliance thereby possibly limiting the growth of smaller healthcare provider and technology organizations. Initiatives to give patients access to their healthcare data, including new proposals from the Center for Medicare and Medicaid Services^22^ are welcome, and in fact it has long been argued that patients themselves should be the owners and guardians of their health data and subsequently consent to their data being used to develop AI solutions.^16^ In this scenario, as in the current scenario where healthcare organizations are the de-facto owners and guardians of patient data generated in the health system alongside fledgling initiatives from prominent technology companies to share patient generated data back into the health system,^23^ there exists the need for secure, high-performance data infrastructure to make use of this data for AI applications.

If the aforementioned issues are addressed, there are two possible routes to building the necessary data infrastructure to enable today’s clinical care and population health management and tomorrow’s AI enabled workflows. The first is an evolutionary path to creating generalized data infrastructure by building on existing impactful successes in the research domain such as the recent Science and Technology Research Infrastructure for Discovery, Experimentation and Sustainability (STRIDES) initiative from the National Institutes of Health^24^ or MIMIC from the MIT Laboratory for Computational Physiology^13^ to generate the momentum for change. Another, more revolutionary path would be for governments to mandate that all healthcare organizations store their clinical data in commercially available clouds. In either scenario, existing initiatives such as the Observational Medical Outcomes Partnership (OMOP^25^) and Fast Healthcare Interoperability Resources (FHIR) standard^26^ that create a common data schema for storage and transfer of healthcare data as well as AI enabled technology innovations to accelerate the migration of existing data^27^ will accelerate progress and ensure that legacy data are included. There are several complex problems still to be solved including how to enable informed consent for data sharing, and how to protect confidentiality yet maintain data fidelity. However, the prevalent scenario for data infrastructure development will depend more on the socio-economic context of the health system in question rather than on technology.

A notable by-product of a move of clinical as well as research data to the cloud would be the erosion of market power of EMR providers. The status quo with proprietary data formats and local hosting of EMR databases favors incumbents who have strong financial incentives to maintain the status quo. Creation of health data infrastructure opens the door for innovation and competition within the private sector to fulfill the public aim of interoperable health data.

The potential of AI is well described, however in reality health systems are faced with a choice: to significantly downgrade the enthusiasm regarding the potential of AI in everyday clinical practice, or to resolve issues of data ownership and trust and invest in the data infrastructure to realize it. Now that the growth of cloud computing in the broader economy has bridged the computing gap, the opportunity exists to both transform population health and realize the potential of AI, if governments are willing to foster a productive resolution to issues of ownership of healthcare data through a process that necessarily transcends election cycles and overcomes or co-opts the vested interests that maintain the status quo—a tall order. Without this however, opportunities for AI in healthcare will remain just that—opportunities.
